# Voluntary Offerings

**Published:** 1894-03-24

**Authors:** 


### THE INSTITUTIONAL WORKSHOP.

### HOSPITAL FINANCE.

### IV.—HOSPITAL SUNDAY AND SATURDAY FUNDS.

The sources of income commonly known as Hospital Sunday and Hospital Saturday Funds are of an importance altogether out of proportion to the percentage which they form of the gross receipts. They have succeeded in bringing the needs of the hospitals face to face with the people, in a manner which had never even been attempted before. It was much in former times if a collection was set aside at rare and irregular intervals for some institution able to bring special influence to bear on the church or chapel in question. By far the greater number of congregations were left from year's end to year's end ignorant of the very needs of the Lazarus at their gate, and of the efforts, continually thwarted for want of money, which were being made by others to relieve him. And this, too, when purse-strings were readily loosened for manifold projects, tending in reality to perpetuate the evils of pauperism, rather than give true assistance to the suffering. The formal recognition by religious communities of the claim of the hospitals on them for support is a revival rather than a novelty, and can be traced back many hundreds of years to the days when the first infirmaries grew up as necessary adjuncts to spiritual aid. But the effort to enlist the sympathies of the workmen for the charities by which they, principally, are benefited, was a purely new experiment. Together, the two Funds are perhaps the most powerful of all engines for maintaining a healthy public interest in the hospitals, and if the amounts collected fluctuate, as they are sure to do, and disappoint the ardour of those foremost in their organisation, they represent, even at their lowest, a larger share in the aggregate of general sympathy and goodwill than has ever before been elicited for this form of charitable work.

The methods of collection in the two Funds are so entirely different that it is necessary to consider them separately. The collection in the case of Hospital Sunday is simplicity itself. Each minister is responsible for the sums contributed through the ordinary church organisation, and for any additional sums which members of the congregation may prefer to forward through his agency. The amounts are distributed by means of the Hospital Committees, or in large towns through the agency of a Special Committee, representative of all the contributing congregations, and the working expenses in the majority of towns are nil, and in no case form a large proportion of the total. The collections on Hospital Sunday furnish 5·28 per cent. of the total income of the general hospitals, and 4·78 per cent. of that of the special hospitals. The proportion is considerably higher in the provincial hospitals than in London. In Scotland the movement has up to the present time taken no hold, but in Ireland it has been found to exercise a marked effect on hospital work, and to have been the means of securing a considerable increase in public interest and support. The amount collected per 1,000 of the population in 1892 varied from £1 and under in certain manufacturing and mining towns, to £25·11 in the case of Lincoln. The amount collected in London was £9·86 per 1,000. It is worth noting that the collections in the individual places of worship for the hospitals rank, in nearly every instance, as by far the largest in the year, showing that the importance of this object over more purely parochial interests is clearly realised. The educative force of the Fund is shown by the increase with some fluctuations year by year in the total amount collected.

The collection of the Hospital Saturday Fund presents many more difficulties, and it would be idle to assert that these difficulties have been yet overcome. The title is perhaps a little misleading. To the outside public it conveys the impression that the Fund exists for the purpose of organising a general street collection on one Saturday in the year. It is indeed by this annual collection that the Fund is brought most forcibly, if not most agreeably, into public notice. It has many inconveniences, among which not the least is the strong temptation to fraud afforded by so indiscriminate a collection, and its success rests on perhaps the weakest of all springs of charity, viz., a man's dislike to say "No" when appealed to for a small sum. Still, as long as London streets continue to be paved with gold (to quote Cardinal Vaughan) to the extent of £5,000 in a day, it will always be deemed worth the picking up, and the enthusiasm of the collectors who are thus drawn into real sympathy with hospital work is a factor well worth reckoning in the sum of good accomplished.

But this street collection is a mere incident in the year's work of the Hospital Saturday Fund. Its *raison d'être* is the support of the artisan, contributed not once and capriciously, but steadily week by week in a box provided for the purpose in the workshop or factory. From this, the vital source of income to the Fund, a sum of £13,745 was derived during the past year. A good idea of the labour involved in the collection of this amount is conveyed in the statement that 8,413 receipts were issued for it, the average amount of these receipts being £1 12s. If we reckon that each of these receipts represents the contributions of ten workmen, paying a penny a week for not less than forty weeks in the year, we have a total of rather over 84,000 subscribers, and something considerably over three million separate donations. In order to attract these donations a complicated process must be gone through, which includes first, gaining the permission of the employer for collections to be made on his premises; second, finding one of the employés to accept the rather thankless task of a weekly application to his fellow-workmen for their pence; thirdly, the provision of properly registered boxes for the offerings; fourthly, the opening of such boxes at stated intervals by an authorised official. All this is not accomplished without a considerable expenditure of labour, much of which is from its exacting and continuous nature out of the scope of voluntary effort. The expenses of such an organisation are inevitably heavy at its commencement, when there is all to do. But every year should now see an improvement in this respect, in which the disproportion between the metropolitan and provincial funds is painfully marked.

In estimating the success of a movement of this nature it is necessary to consider it under two widely differing aspects. From one point of view it is a source of income to the hospitals. From another it is a workmen's provident association for obtaining medical relief. The example of Birmingham and of the Potteries has shown that there is nothing antagonistic in the two theories. Charity may well go hand in hand with prudence. But any confusion in practice of the one with the other results in a serious weakening of the force of both. There is evidence in Mr. Acland's annual address on behalf of the Metropolitan Fund that its promoters are keenly alive to this difficulty. The proposal to strengthen the provident element of the Fund by an alliance with the Provident Medical Association is one which should meet with earnest and impartial consideration at the hands of all its friends. That the Fund is administered practically for the benefit of those who contribute to it is emphasised by the fact that no fewer than 24,896 letters of recommendation were issued to hospitals and homes by the Distribution Committee during the past year. If these letters were not mainly bestowed upon contributing workmen and their families, on what grounds does the Fund burden itself with a responsibility which might far better fall on local agencies of relief?

We believe that nothing but good can come from emphasising the provident nature of the work carried on by the Hospital Saturday Funds all over England. To relieve the injustice to medical men, and the strain on the hospitals inflicted by unsuitable admissions, is an important part of this work, and one which the Provident Medical Association aims mainly at achieving. To make clear to the workmen of each small contributing centre what benefits they and their families receive from the Fund is the next important step. This has in many places been managed with great success and small trouble by the system of hospital letters of introduction books issued to each subscribing workshop, but whatever the form of machinery adopted the balance between giving and taking should be accurately defined to the contributors.

The charitable aspect of the Fund, far from being obscured, will tend to come even more prominently into relief when disentangled from the purely prudential element. Among the poorer classes, in London at any rate, the spirit of generosity is far more easily evoked than the spirit of thrift. Let the workman know what proportion of contributions is presented as a free gift to the hospitals and the noble spirit of emulation in charity which has produced such good results in many provincial towns and in congregational collections, will stir him to a sustained habit of liberality and place our hospitals on a sounder, because broader basis of prosperity than they have ever hitherto known.

